# Inclusion of persons with disabilities in systems of social protection: a population-based survey and case–control study in Peru

**DOI:** 10.1136/bmjopen-2016-011300

**Published:** 2016-08-25

**Authors:** Antonio Bernabe-Ortiz, Francisco Diez-Canseco, Alberto Vasquez, Hannah Kuper, Matthew Walsham, Karl Blanchet

**Affiliations:** 1 CRONICAS Centre of Excellence in Chronic Diseases, Universidad Peruana Cayetano Heredia, Lima, Peru; 2 Department of Epidemiology and Population Health, London School of Hygiene and Tropical Medicine, London, UK; 3 School of Medicine, Universidad Peruana de Ciencias Aplicadas—UPC, Lima, Perú; 4 Sociedad y Discapacidad—SODIS, Lima, Perú; 5 International Centre for Evidence in Disability, London School of Hygiene and Tropical Medicine, London, UK

**Keywords:** EPIDEMIOLOGY, PUBLIC HEALTH, SOCIAL MEDICINE

## Abstract

**Objective:**

This study aims to assess the needs of people with disabilities and their level of inclusion in social protection programmes.

**Design:**

Population based-survey with a nested case–control study.

**Setting:**

Morropon, a semiurban district located in Piura, northern Peru.

**Participants:**

For the population survey, a two-stage sampling method was undertaken using data from the most updated census available and information of each household member aged ≥5 years was collected. In the nested case–control study, only one participant, case or control, per household was included in the study.

**Primary and secondary outcome measures:**

Disability was screened using the Washington Group short questionnaire. A case, defined as an individual aged ≥5 years with disabilities, was matched with one control without disabilities by sex and age (±5 years). Information was collected on socioeconomic status, education, health and rehabilitation and social protection participation.

**Results:**

The survey included 3684 participants, 1848 (50.1%) females, mean age: 36.4 (SD: 21.7). A total of 290 participants (7.9%; 95% CI 7.0% to 8.7%) were classified as having disability. Adults with disabilities were more likely to be single (OR=3.40; 95% CI 1.54 to 7.51) and not to be working (OR=4.36; 95% CI 2.26 to 8.40), while those who did work were less likely to receive the national minimum wage (ie, 750 PEN or about US$265; p=0.007). People with disabilities were more likely to experience health problems. There was no difference between those enrolled in any social protection programme among participants with and without disabilities.

**Conclusions:**

People with disabilities were found to have higher needs for social protection, but were not more likely to be enrolled in social protection programmes. The Peruvian social protection system should consider adding disability status to selection criteria in their cash transfer programmes as well as implementing disability-specific interventions.

**Strengths and limitations of this study**

- This study included two different methodological designs to assess the prevalence of, and factors associated with disability in a district in Peru.
- Social protection systems may be important vehicles to promote the social inclusion of people with disabilities, but there is limited information regarding their impact on disability in resource-constrained settings.
- Local poverty conditions of semiurban settings can hide some of the well-known gaps related to participation and social inclusion of people with disabilities.

## Introduction

As described by the United Nations Convention on the Rights of Persons with Disabilities, “persons with disabilities include those who have long-term physical, mental, intellectual or sensory impairments which in interaction with various barriers may hinder their full and effective participation in society on an equal basis with others”.^1^ Worldwide, over a billion persons live with a disability, and about 110–190 million adults have very significant difficulties in functioning.^2^ Moreover, in the past two decades, the number of persons living with disabilities has increased due to population growth and ageing.^3^

Social protection, consisting of policies and programmes to provide basic income security and access to essential social services, has become important on the international agenda.^4^ Through social protection programmes, poverty, vulnerabilities and livelihood protection are addressed, often focusing on people who are particularly vulnerable as a result of life cycle (old age), economic characteristics (unemployment), health risks (sickness) or natural and ecological factors.^5^ As a result, social protection can serve to alleviate poverty and enhance living conditions of the target population.

In Peru, social protection has been placed as one of the main priorities of the current government.^5^ Different types of social protection programmes are implemented. One type is cash transfer programmes, such as ‘Juntos’ and ‘Pension 65’. ‘Juntos’ is focused on households with children under 18 and pregnant women living in poverty or extreme poverty. Conditional on education, nutrition and health requirements, families receive a monthly incentive of 100 PEN (about US$35 at the time of the study).^6^ On the other hand, ‘Pension 65’ is for older adults (≥65 years) who live in extreme poverty.^7^ Health insurance schemes are also offered in Peru, such as the ‘Seguro Integral de Salud’ (Comprehensive Health Insurance, SIS), which aims to protect Peruvians’ health if they are not enrolled in health insurance schemes, prioritising vulnerable populations in extreme poverty.^8^

Evidence shows that people with disabilities face barriers to many core social activities and services targeted by social protection projects, including health and rehabilitation, education, livelihoods and political participation.^9–11^ Overcoming these difficulties requires interventions to reduce environmental and social barriers as well as addressing the needs related to their impairments. Social protection systems may therefore be important vehicles to promote the social inclusion of people with disabilities, especially those related to education, employment and health, whether by including people with disabilities in mainstream programmes or by developing programmes specifically for people with disabilities.

Therefore, the aim of this study was to assess the access of people with disabilities to education, employment and health as well as their needs for and inclusion in social protection in comparison to people without disabilities.

## Methods

### Study setting

Morropon, a semiurban district located in Piura, northern Peru, was selected as the study setting. Morropon experiences high rates of poverty, with ∼80% of the participants living there being considered as poor or extremely poor.^12^ Consequently, we anticipated that a high proportion of participants would be eligible and/or enrolled in social protection programmes, allowing us to examine their relationship with disability. At the time of the study, 1409 households were enrolled in the ‘Juntos’ programme and 569 individuals were included in the ‘Pension 65’ programme.^13^ According to the 2007 National Census,^14^ Morropon has 14 421 inhabitants, of whom 15% are illiterate and 48.7% do not have health insurance. The prevalence of disability was estimated at 4.5% according to the National Survey on Disability (Encuesta Nacional Especializada sobre Discapacidad, ENEDIS) performed in 2012.^15^

### Study phases

The study comprised two phases: a disability survey in the general population (phase 1) and the assessment of access to education, employment and health, as well as needs for social protection (phase 2).

### Phase 1: disability survey in the general population

A population-based survey was conducted to estimate the prevalence of disability and to assess the inclusion in selected social protection programmes (‘Juntos’, ‘Pension 65’ and ‘Seguro Integral de Salud’) by disability status.

A two-stage sampling method was undertaken using data and maps from the most updated census available (2007 National Census). First, a random sample of 90 clusters was selected across the district, each comprising one individual residential block and containing, on average, 20–40 households. In the second stage, the households were visited door to door and the head of the family or his/her spouse was interviewed to collect data on the household composition and the age, sex and disability status of each household member aged 5 years and above. Disability was defined using the Washington Group Short Set of Questions on Disability, translated into Spanish.^16^ The tool includes six questions about difficulties with activities (seeing, hearing, walking or climbing stairs, remembering or concentrating, self-care, and communicating) as a result of a health problem. These questions were rated by the responder using four options: no difficulty, some difficulty, a lot of difficulty or cannot do at all. Accordingly, a participant responding ‘cannot do at all’ or ‘a lot of difficulty’ to any of the six questions as well as those who reported some difficulty in at least two questions was considered to have a disability. In addition, the household informant also provided household-level data on social protection programmes access (‘Juntos’ and ‘Pension 65’), family income and household characteristics including assets and access to services.

### Phase 2: assessment of exclusion and needs for social protection

A case–control study was performed by selecting people with disabilities (‘cases’) identified in phase 1. A case was defined as a male or female, aged 5 years and above, with disabilities based on the Washington Group screening questions.^16^ A control (ratio case–control: 1:1) of the same sex and similar age (±5 years), but without disabilities, was selected from the survey population of the same cluster. Only one participant, case or control, per household was included in this phase of the study.

### Questionnaire

The cases and controls were interviewed in detail using a semistructured questionnaire. They were asked again about disability status using the Washington Group short set of questions, if a proxy response had been used in the initial screening. In addition, the questionnaire included modules on sociodemographics (sex, age, ethnicity, familial income and socioeconomic status), education (schooling), employment, (work and income), access to health (health insurance), and areas pertinent to social protection programmes (cash transfer as well as access to other programmes, such as SIS), so as to compare the living circumstances of persons with disabilities to those without. All these items were drawn from previously used questionnaires in Peru and elsewhere and were based on widely used items.^15^ ^17^ Although the tools were not validated for this setting, they were pilot-tested to evaluate their performance in the fieldwork. All questionnaires were administered in Spanish by trained fieldworkers.

### Data analysis

Data were analysed using STATA V.13 for Windows (Stata Corp, College Station, Texas, USA). Gender-stratified estimations of prevalence of disability and types of impairments in the general population were performed. Comparisons of disability prevalence within social protection programmes were also tabulated. Data were analysed at the household and individual levels for ‘Juntos’ and ‘Pension 65’ membership, respectively.

Owing to age-matching and gender-matching, comparisons between cases and controls were performed using McNemar's χ^2^ test or simple χ^2^ test when appropriate. These tests were created to identify differences between cases and controls in the domains of education, employment and health as well as areas pertinent to the social protection programmes. Female-specific analysis was performed to compare cases and controls among women of reproductive age (15–49 years), for instance, for reproductive health-related variables.

### Ethics

Written informed consent from each participant and/or caregiver was obtained before starting fieldwork activities.

## Results

### Phase 1: disability survey in the general population

#### Description of the study population

A total of 1128 households were invited to take part in the study, of which 44 (3.9%) refused to participate. Thus, 4021 participants in the 1084 households were recorded; of them, 335 (8.3%) were excluded from further analyses because their age was under 5 years. Thus, 3684 participants, 1848 (50.1%) females, mean age: 36.4 (SD: 21.7), were assessed. Most of the study population (53.3%) reported a monthly family income <450 PEN (US$158).

According to the study criteria, 290 participants (7.9%; 95% CI 7.0% to 8.7%) were classified as having a disability. The most common domain was difficulty walking (2.4%), followed by difficulty seeing (2.1%). See details in online supplementary E-table S1. At the family level, 188 (17.3%) families included one person with disabilities, whereas 39 (3.6%) and 8 (0.7%) families included two and three persons with disabilities, respectively.

10.1136/bmjopen-2016-011300.supp1 Supplementary E-table

#### Disability, sociodemographic factors and social protection programmes

There was no difference in the prevalence of disability between males and females; however, it was higher among older individuals compared to younger persons (table 1). There was a clear inverse relationship between familial income and prevalence of disability, such that people in the lowest quartile of income were more than 7 times more likely to have a disability. Similarly, those in the poorest quartile of socioeconomic status were more than three times more likely to have a disability.

**Table 1:** Survey of the general population: sociodemographic characteristics according to disability

	People with disabilities/total	Prevalence of disability (95% CI)	OR (95% CI)
Gender			
Male	135/1786	7.6% (6.3% to 8.8%)	1 (Reference)
Female	152/1787	8.5% (7.2% to 9.8%)	1.15 (0.91 to 1.47)
Age categories (years)			
5–9	12/341	3.5% (1.6% to 5.5%)	1 (Reference)
10–19	19/749	2.5% (1.4% to 3.7%)	0.71 (0.34 to 1.49)
20–29	14/465	3.0% (1.5% to 4.6%)	0.85 (0.39 to 1.86)
30–39	17/465	3.7% (1.9% to 5.4%)	1.04 (0.49 to 2.21)
40–59	47/943	5.0% (3.6% to 6.4%)	1.44 (0.75 to 2.74)
60+	178/610	29.2% (25.6% to 32.8%)	**11.30 (6.19 to 20.62)**
Familial income			
Up to 100 PEN	19/85	22.4% (13.3% to 31.4%)	1 (Reference)
101–450 PEN	167/1730	9.7% (8.3% to 11.0%)	**0.37 (0.22 to 0.63)**
451–750 PEN	55/959	5.7% (4.3% to 7.2%)	**0.21 (0.12 to 0.38)**
751+ PEN	25/642	3.9% (2.4% to 5.4%)	**0.14 (0.07 to 0.27)**
Socioeconomic status*			
1st quartile (poorest)	116/888	13.1% (10.8% to 15.3%)	1 (Reference)
2nd quartile	67/871	7.7% (5.9% to 9.5%)	**0.54 (0.40 to 0.74)**
3rd quartile	57/910	6.3% (4.7% to 7.8%)	**0.45 (0.32 to 0.62)**
4th quartile (wealthiest)	47/904	5.2% (3.7% to 6.6%)	**0.37 (0.26 to 0.52)**

Bold estimates are statistically significant (p<0.05).

*Socioeconomic status was evaluated by creating a wealth index based on household assets and then split into quartiles.

A total of 356/1082 (32.9%) families reported being enrolled in ‘Juntos’. Among families with at least one member with disabilities, 67/235 (28.5%) were in the programme, whereas enrolment was higher among families without members with disabilities (289/847: 34.1%) (p=0.11). Conversely, when focusing on the 28 families with a child with disabilities, 24 (85.8%) were enrolled in ‘Juntos’, which is higher than the proportion (515/926: 55.6%) among families with children without disabilities (p=0.002).

Only elderly participants (65 years and above) were considered in the analyses of the ‘Pension 65’ cash transfer programme. There was no difference between those enrolled in ‘Pension 65’ among participants with (31.8%) and without disabilities (23.9%; p=0.07).

Finally, more than three quarters of the study sample were enrolled in the SIS with no difference between those with (75.5%) or without disabilities (79.0%; p=0.16).

### Phase 2: assessment of access and needs for social protection

Out of 290 subjects with disabilities screened in the Phase 1 of the study, 55 lived in the same household with another person with disability, 35 cases had no control available, and 39 cases refused to participate or were unavailable. Therefore, only 161 cases with disabilities were matched by sex and age with 161 controls. Overall, cases had a mean age of 56.8 (SD: 24.2) years, compared to controls aged 56.1 (SD: 23.7) years.

### Social protection needs and disability in adults aged ≥18 years

One hundred and forty-one cases and their respective controls were aged 18 years and above. Details of the distribution of sociodemographic characteristics are presented in table 2. Of note, being single, self-reported Caucasian/white ethnicity and illiteracy were associated with greater odds of being persons with disabilities, while not having attended school was weakly associated with disability.

**Table 2:** Case–control study: association between disability and sociodemographic characteristics in adults aged ≥18 years

	Cases (n=141)	Controls (n=141)	Conditional OR
*Sociodemographics*			
Gender			
Female (vs male)	81 (57.5%)	81 (57.5%)	–
Age categories (years)			
18–29	8 (5.7%)	8 (5.7%)	–
30– 49	24 (17.0%)	24 (17.0%)	–
50–69	45 (31.9%)	50 (35.5%)	–
70+	64 (45.4%)	59 (41.8%)	–
Marital status			
Married/cohabiting	66 (46.8%)	84 (59.6%)	1 (Reference)
Divorced/separated/widowed	42 (29.8%)	43 (30.5%)	1.07 (0.55 to 2.06)
Single	33 (23.4%)	14 (9.9%)	**3.40 (1.54 to 7.51)**
Ethnicity			
Mestizo (Amerindian)	92 (69.7%)	116 (84.7%)	1 (Reference)
African-Peruvian/black	12 (9.1%)	9 (6.6%)	1.61 (0.63 to 4.08)
Caucasian/white	28 (21.2%)	12 (8.8%)	**2.61 (1.25 to 5.48)**
*Schooling*			
School attendance			
No (vs yes)	37 (26.2%)	24 (17.0%)	1.72 (0.96 to 3.08)
Highest academic attainment			
Up to incomplete primary	53 (51.0%)	60 (51.3%)	1 (Reference)
Complete primary/basic	19 (18.3%)	15 (12.8%)	1.49 (0.55 to 4.03)
Incomplete/complete secondary	20 (19.2%)	27 (23.1%)	0.82 (0.32 to 2.15)
Superior or more	12 (11.5%)	15 (12.8%)	0.60 (0.18 to 1.98)
Literacy			
Good	50 (35.5%)	64 (45.4%)	1 (Reference)
Not so good	45 (31.9%)	54 (38.3%)	1.14 (0.62 to 2.07)
Illiterate (cannot read)	46 (32.6%)	23 (16.3%)	**2.71 (1.38 to 5.32)**
*Employment*			
Worked in the past 7 days?			
No (vs yes)	109 (77.3%)	71 (50.4%)	**4.45 (2.32 to 8.57)**
Worked in the past year?			
No (vs yes)	105 (74.5%)	68 (48.2%)	**4.36 (2.26 to 8.40)**
*Health*			
Enrolled in health insurance			
No (vs yes)	24 (17.0%)	27 (19.2%)	0.85 (0.45 to 1.62)
Past 12 months, health problems			
No	18 (12.8%)	52 (36.9%)	1 (Reference)
Yes, but not serious	28 (19.8%)	42 (29.8%)	1.72 (0.75 to 3.92)
Yes, and serious	95 (67.4%)	47 (33.3%)	**5.69 (2.78 to 11.65)**

Bold estimates are statistically significant (p<0.05).

– Not calculable since age and sex were the matching variables.

Compared to controls, people with disabilities were over four times more likely not to have worked in either the past 7 days or the past year. Although the main reason for unemployment (i.e. being older) was the same among cases (44.1%) and controls (61.5%); cases with disabilities reported having physical limitations (21.6%) and having chronic conditions (18.6%) as other important reasons for not working. Secondary reasons among controls included caring for children (27.7%) and not finding work positions (4.6%). Among those who reported working, only income but not job type or form of payment was significantly different: 87% of cases received <400 PEN (US$∼140) monthly, whereas it was true for only 54.4% of controls (p=0.007).

Self-report of serious health problems were more frequent among cases (67.4%) than controls (33.3%, p value <0.001); however, people with disabilities were not more likely to be enrolled in health insurance schemes, with enrolment rates of above 80% for both groups (p=0.64). Among those who reported having a health problem (123 cases and 89 controls), 61% of cases and 64% of controls reported always seeking healthcare, 26% of cases and 30% of controls reported seeking healthcare sometimes and 13% of cases and 5.6% of controls reported never seeking healthcare (p=0.20). Cases mainly sought treatment in health centres (p<0.001) compared to controls who sought treatment in pharmacies (p=0.003).

Only 19 cases and 19 controls were included who were women aged 15–49 years. Among these, only 7 (36.8%) cases reported having children compared to 16 (88.9%) controls (p=0.002). Only nine women reported having previously had an abortion or miscarriages (3 cases and 6 controls, p=0.46). All women who reported having a pregnancy in the previous 5 years also reported having accessed prenatal care (1 case and 10 controls) and in all cases the birth was attended to by a midwife. All the children born in this period have received vaccines (1 case and 10 controls).

### Access, social protection needs and disability in adults aged <18 years

Twenty children with disabilities and their respective controls were included in the analyses. Characteristics of cases and controls are shown in table 3. Of note, more than a quarter of children with disabilities were one grade lower than controls (26.5% vs 5.0%, respectively), although this difference was not significant (p=0.09). Regarding health, 18 (90.0%) cases reported having a health problem in the past 12 months (60.0% serious) compared to 12 (60.0%) controls (only 10.0% serious, p=0.03).

**Table 3:** Case–control study: association between inclusion and disability in children aged <18 years

	Cases (n=20)	Controls (n=20)	Conditional OR
*Sociodemographics*			
Gender			
Female (vs male)	9 (45.0%)	9 (45.0%)	–
Age (years)			
5–7	2 (10.0%)	3 (15.0%)	–
8–11	9 (45.0%)	8 (40.0%)	–
12–17	9 (45.0%)	9 (45.0%)	–
*Schooling*			
Currently enrolled at school			
No	1 (5.0%)	0 (0.0%)	–
Same grade as other children			
Yes	14 (73.7%)	19 (95.0%)	1 (Reference)
No, one grade below	5 (26.3%)	5 (5.0%)	5.0 (0.58 to 42.8)
Days of school missed			
None	15 (79.0%)	19 (95.0%)	1 (Reference)
1+ days	2 (10.5%)	0 (0.0%)	4.0 (0.45 to 35.8)
Highest academic attainment			
Complete/incomplete primary	13 (68.4%)	12 (60.0%)	1 (Reference)
Complete/incomplete secondary	6 (31.6%)	8 (40.0%)	0.5 (0.05 to 5.51)
Ever repeated a school year			
Yes (vs no)	5 (26.4%)	2 (10.0%)	4.0 (0.45 to 35.8)
*Health*			
Enrolled in health insurance			
Yes (vs no)	19 (95.0%)	20 (100.0%)	–
Past 12 months, health problems			
No	2 (10.0%)	8 (40.0%)	1 (Reference)
Yes, but not serious	6 (30.0%)	10 (50.0%)	1.51 (0.25 to 9.17)
Yes, and serious	12 (60.0%)	2 (10.0%)	**13.8 (1.37 to 138.1)**

Bold estimates are statistically significant (p<0.05).

– Not calculable. These estimates cannot be calculated for age and sex, since these were the matching variables, or for school enrolment (100% among controls) or health insurance enrolment (100% among controls).

### Specialised health and assistive device needs

**Figure 1:**
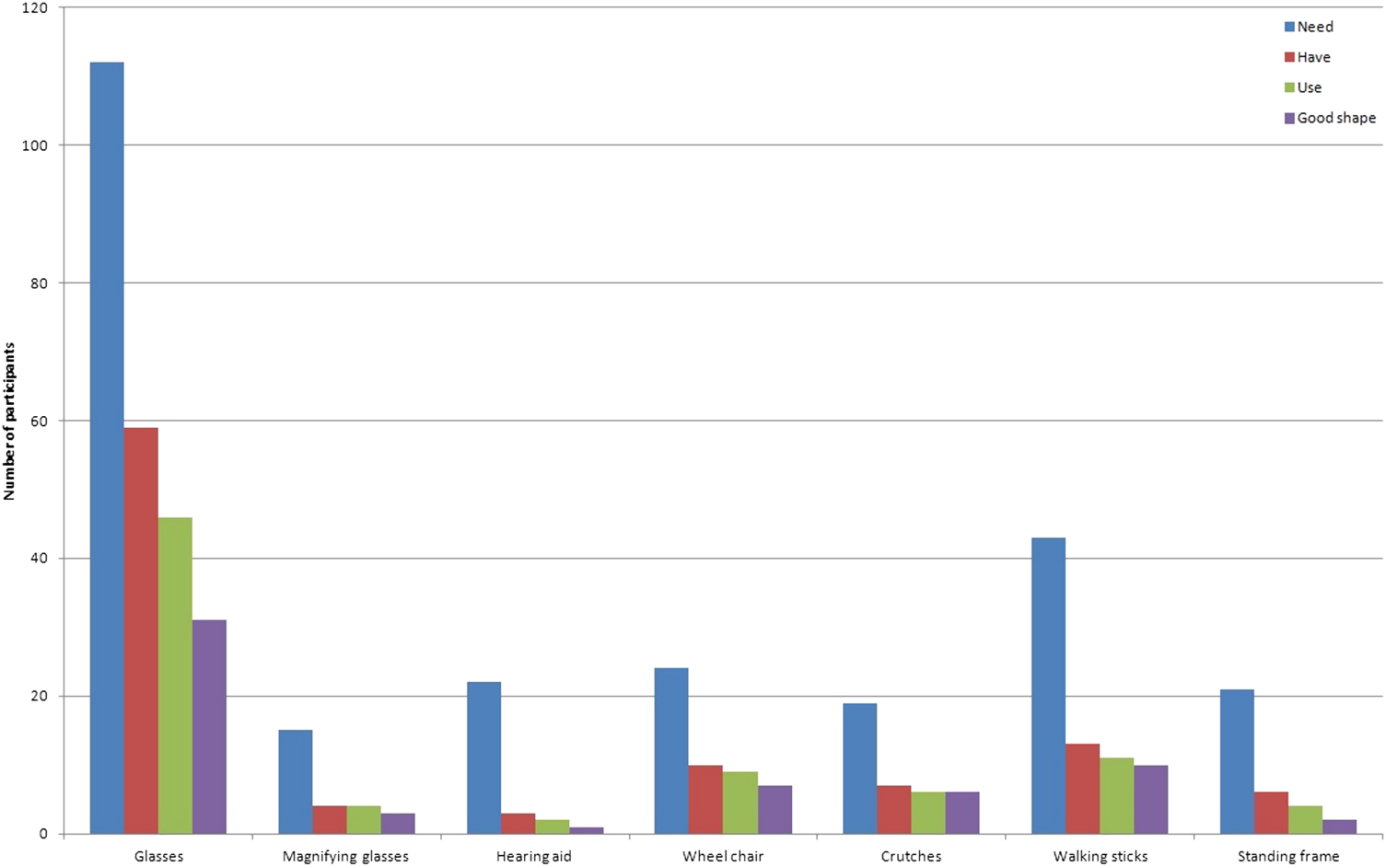
Assistive devices need, ownership and use among cases with disabilities. Only people with disabilities (cases) were included in the analysis.

Cases (n=161) were asked about access to specialised health and needs for assistive devices (figure 1). From the health perspective, 106 (65.8%) had heard about rehabilitation services, 140 (86.9%) had heard about specialised health services and only 51 (31.7%) had heard about assistive devices. Only 5% (3/60) of those who needed rehabilitation reported using the service, whereas it was the case for 18.6% (22/118) in specialised health service. From the counselling perspective, 149 (92.5%) participants with disabilities reported having heard about healers, whereas 37.3% reported having heard about familial counselling or special education services. Only 15% (6/40) reported using healers, 38.9% (7/18) reported using familial counselling and 33.3% (2/6) reported using special education services.

The need for assistive devices was also assessed among persons with disabilities. Glasses were reported as needed by 112 (69.6%), followed by walking sticks (n=43; 26.7%) and wheel chairs (n=24, 14.9%); 85.7% reported that they were not aware of the Braille system, and this proportion was also high for awareness regarding recorders (80.8%), enlarged prints (74.5%), guides (67.7%), white canes (51.6%) and prostheses (50.9%). Only five participants reported needing prostheses, whereas two needed enlarged prints and one needed the Braille system. Moreover, no one reported needing a recorder, white cane or guide.

## Discussion

### Main findings

Disability was common in this Peruvian semiurban setting—almost 8% of the population aged 5 years and above reported having a disability and one in six households included a person with a disability. The prevalence of disability did not vary by gender, but was higher among older people, those with lower familial income and those in the lowest socioeconomic status. People with disabilities in this area appeared to have a greater need for social protection. This is indicated by the fact that the adults with disabilities were more likely to be single and not to be working, while those who did work were less likely to receive the national minimum wage. Furthermore, both adults and children with disabilities experienced significantly more frequent serious health conditions and had low access to and use of specialist health services including medical rehabilitation and assistive device services. However, our results suggest no differences in the inclusion of people with disabilities compared to those without disabilities in the selected social protection programmes, despite their greater vulnerability and need.

### Comparison with the literature

Prevalence of disability varies between countries, especially depending on the tools used in the evaluation.^18–23^ A report using the Washington Group's questions in Mexico reported 5.1%,^24^ whereas an estimation of disability for 54 countries reported 14%.^25^ Our results are higher than those reported by a national survey in Peru,^17^ although an adaptation of the Washington Group questions were used to define disability as a permanent limitation. When the definition of disability was restricted to those reporting a lot of or total difficulty, prevalence reduced to 5.6% in our study, a result comparable to estimates from the national survey.

Older age, poverty and unemployment were factors associated with greater disability prevalence.^26^ Disability was, however, similar between males and females, in contrast to some reports showing greater disability among women.^2^ ^26^ As in previous reports, persons with disabilities tend to be of lower socioeconomic status and to be concentrated in poorer areas and to be less likely to work.^18^ Moreover, extra costs of disability should be also considered. Usually, persons with disabilities incur a range of common daily expenditures that persons without disabilities do not. Extra costs include, but are not limited to, transportation, personal assistance, healthcare, assistive devices and, in some cases, house adaptation,^27^ ^28^ and these are usually not covered by social protection programmes. As a result, one could argue that the eligibility threshold for means-tested programmes should be lower for households that include people with disabilities. The insufficiency of social protection programmes among people with disabilities may therefore be even higher than our estimation.

Independence and quality of life of persons with disabilities improves with the use of assistive devices,^29^ including the psychosocial impact for the user.^30^ However, we found that access to and use of specialist health services including medical rehabilitation and assistive device services were very low in our study setting. Evidence suggests that people with disabilities have great unmet needs because of increased costs and a range of barriers when they attempt to access healthcare,^26^ ^31^ and also because of inadequate health worker skills and the absence of specialised health services.^10^ ^32^ The same case applies for specific device needs, including a lack of knowledge regarding the existence of technologies and devices that can maximise functioning. As the WHO reported for resource-constrained settings,^2^ <15% of people who require assistive devices are able to access them. Thus, current health service provision is inadequate to meet the complex needs of persons with disabilities.^32^

There is a lack of evidence on the inclusion of people with disabilities into social protection programmes in low-income and middle-income countries with which we can compare our findings.^33^ However, existing studies suggest that barriers faced by people with disabilities include inaccessible administrative offices and service providers, discrimination by programme staff, problems complying with conditions attached to benefits and limited awareness of entitlements among people with disabilities themselves.^34^ The failure to take the higher expenditures people with disabilities face and their particular needs (eg, accessing health and education services) may limit the impact of programmes in addition to their levels of access.^35^ ^36^

### Implications

The inclusion of people with disabilities is important within the context of social protection, as they are poorer and less likely to be working in our study and in previous reports.^37^ This is in part because disability is more common in already vulnerable groups. Additionally, people with disabilities face restrictions to their inclusion and participation in society as a result of social and contextual factors, which can reduce access to education, employment and healthcare and the full realisation of their human rights.^26^

‘Juntos’, a familial social protection programme, is a conditional cash transfer programme to reduce poverty. Participants acquired a list of commitments to improve access to education and health.^6^ ‘Pension 65’, another cash transfer programme, is focused on the elderly without appropriate resources of subsistence.^7^ It is encouraging to see equal access among families/individuals with and without disabilities in social protection programmes, but coverage of both programmes is relatively low as only 28.5% of families with a member with disabilities are enrolled in ‘Juntos’ and 31.8% of the elderly with disabilities in ‘Pension 65’. Since these programmes are not specific for people with disabilities, low coverage may be related to disability not being among the criteria for enrolment. In addition, existing social protection programmes might be insufficient to appropriately cover people with disabilities as extra costs due to disability are present.^38^ Moreover, the presence of a person with disability in the family might reduce the other member's ability to work. This potentially can be another pathway to understand the link between poverty and disability.^39^ ^40^ On the other hand, more than three quarters of participants were enrolled in the SIS programme. Since disability is seen as a consequence of health impairment, the gap among people with disabilities exists but is low compared to the cash transfer programmes. This should be reconsidered in the light of this study's findings related to increased health problems and decreased livelihood opportunities among people with disabilities.

### Strengths and limitations

This is a detailed study including two different methodological designs to assess the prevalence as well as factors associated with disability in a district in Peru. The main strengths include the enrolment of participants from all ages, as well as the assessment of social protection programmes. Nevertheless, some limitations deserve consideration. The design of the study can only determine association and not causality, which can be important for a more detailed ascertainment of care and service needs. Moreover, Morropon is a poor region within Peru, and so the results may not be generalisable to other settings in the country. However, social protection programmes in Peru are implemented in similar regions (poor and extremely poor areas) and are implemented in a similar way. Therefore, the results might have relevance for other areas in Peru. The short set of questions of the Washington Group was used, and for that reason cognition and mental functioning were not adequately addressed. In addition, the questionnaire measures used in the study were not validated for the study setting. However, this was pilot-tested before application. The six dimensions of the Washington Group questions were grouped, which unfortunately did not allow for comparisons between different types of impairments. Although we tried to show information regarding gender and child age, the sample size was not large enough to demonstrate differences between groups. Besides, we did not measure the extra costs associated with disability, which would most likely have further increased the unmet need for social protection. Finally, owing to the selection of a semiurban setting, local poverty conditions can hide some of the well-known gaps related to participation and social inclusion of people with disabilities. However, our findings in combination suggest greater economic vulnerability for people with disabilities and greater need for social support to guarantee appropriate inclusion.

### Conclusions

Disability is a common condition in Peru. People with disabilities have a greater need for social protection but this is not reflected in higher levels of social protection enrolment. The Peruvian social protection system should consider adding disability status to selection criteria in their mainstream cash transfer programmes as well as implementing disability-specific interventions.
