# Supplementary material for: Inclusion of persons with disabilities in systems of social protection: a population-based survey and case–control study in Peru
Source: BMJ Open. 2016 Aug 25;6(8):e011300. doi: 10.1136/bmjopen-2016-011300 (PMC5013477; doi:10.1136/bmjopen-2016-011300)
Supplement: Supplementary E-table [file bmjopen-2016-011300supp_table.pdf]

ONLINE SUPPLEMENT

E-Table 1: Domains of disability among males, females and total population

Difficulty with...	Total population screened	Male population screened	Female population screened
Seeing, n (%)			
No	3,422 (92.1)	1,727 (93.3)	1,695 (90.9)
Some	217 (5.8)	86 (4.7)	131 (7.0)
A lot	63 (1.7)	32 (1.7)	31 (1.7)
Cannot do at all	13 (0.4)	6 (0.3)	7 (0.4)
Hearing, n (%)			
No	3,545 (95.7)	1,761 (95.2)	1.784 (96.0)
Some	108 (2.9)	58 (3.1)	50 (2.7)
A lot	46 (1.2)	26 (1.4)	20 (1.1)
Cannot do at all	7 (0.2)	4 (0.2)	3 (0.2)
Walking, n (%)			
No	3,525 (95.2)	1,776 (96.2)	1,749 (94.2)
Some	89 (2.4)	32 (1.7)	57 (3.1)
A lot	67 (1.8)	29 (1.6)	38 (2.0)
Cannot do at all	23 (0.6)	10 (0.5)	13 (0.7)
Remembering, n (%)			
No	3,538 (95.5)	1,776 (96.2)	1,762 (94.8)
Some	101 (2.7)	44 (2.4)	57 (3.1)
A lot	50 (1.4)	21 (1.1)	29 (1.6)
Cannot do at all	14 (0.4)	5 (0.3)	9(0.5)
Self-care, n (%)			
No	3,625 (97.8)	1,818 (98.4)	1,807 (97.4)
Some	24 (0.7)	9 (0.5)	15 (0.8)
A lot	26 (0.7)	9 (0.5)	17 (0.9)
Cannot do at all	28(0.8)	11 (0.6)	17 (0.9)
Communicating, n (%)			
No	3,616 (97.6)	1,800 (97.5)	1,816 (97.9)
Some	50 (1.4)	26 (1.4)	24 (1.3)
A lot	25 (0.7)	15 (0.8)	10 (0.5)
Cannot do at all	11 (0.3)	5 (0.3)	6 (0.3)
